# Gastroscopic Image Graph: Application to Noninvasive Multitarget Tracking under Gastroscopy

**DOI:** 10.1155/2014/974038

**Published:** 2014-08-24

**Authors:** Bin Wang, Weiling Hu, Jiquan Liu, Jianmin Si, Huilong Duan

**Affiliations:** ^1^College of Biomedical Engineering & Instrument Science, Zhejiang University, Hangzhou, Zhejiang 310027, China; ^2^Key Laboratory for Biomedical Engineering, Ministry of Education, Hangzhou, Zhejiang 310027, China; ^3^Department of Gastroenterology, Sir Run Run Shaw Hospital, Zhejiang University, Hangzhou, Zhejiang 310016, China

## Abstract

Gastroscopic examination is one of the most common methods for gastric disease diagnosis. In this paper, a multitarget tracking approach is proposed to assist endoscopists in identifying lesions under gastroscopy. This approach analyzes numerous preobserved gastroscopic images and constructs a gastroscopic image graph. In this way, the deformation registration between gastroscopic images is regarded as a graph search problem. During the procedure, the endoscopist marks suspicious lesions on the screen and the graph is utilized to locate and display the lesions in the appropriate frames based on the calculated registration model. Compared to traditional gastroscopic lesion surveillance methods (e.g., tattooing or probe-based optical biopsy), this approach is noninvasive and does not require additional instruments. In order to assess and quantify the performance, this approach was applied to stomach phantom data and *in vivo* data. The clinical experimental results demonstrated that the accuracy at angularis, antral, and stomach body was 6.3 ± 2.4 mm, 7.6 ± 3.1 mm, and 7.9 ± 1.6 mm, respectively. The mean accuracy was 7.31 mm, average targeting time was 56 ms, and the *P* value was 0.032, which makes it an attractive candidate for clinical practice. Furthermore, this approach provides a significant reference for endoscopic target tracking of other soft tissue organs.

## 1. Introduction

Gastroscopic multitarget tracking can serve to advance the surgical field by reducing the invasiveness of procedures. For example, an endoscopist could mark suspicious lesions during a gastroscopy, and the marked lesions could be tracked and diagnosed in the follow-up surgery [1–3]. Furthermore, during the process of * in vivo* evaluation and therapy planning, the preobserved lesion marked by the tracking approach can be considered a navigation scheme to target biopsy sites. This facilitates improvement of the precision of the biopsy targeting and the validity of lesion analysis [4–6].

The traditional target tracking approach for endoscopy is tattooing [7, 8]. During the tattooing procedure, the endoscopist injects Indian ink into the lesion sites or marks the lesion sites with argon plasma coagulation (APC) [9, 10]. In the follow-up examination, the tattooing markers guide the endoscopist to track and retarget the lesion sites. Unfortunately, this approach has its disadvantages. (1) Ink may fade over time after several months. (2) Inking and APC are invasive operations and damage the tissue. (3) The marker created by ink and APC is procedurally cumbersome and carries a risk of technical failure.

To overcome these disadvantages, many papers in the literature have investigated target tracking techniques that utilize novel approaches. Allain et al. introduced a biopsy tracking method based on epipolar geometry and evaluated the results' uncertainty with a covariance matrix [11]. However, since the accuracy of using epipolar geometry as an analytical tool depends largely on the view angle, this method is not viable for a gastroscopy procedure. Helferty et al., Vining et al., and Zhang et al. took advantage of virtual endoscopy to analyze and track lesions [12–14], but the virtual endoscopy required extra CT or MRI scans, which increased the operation expense. Moreover, the preoperative static model could not represent the stomach's deformation motion accurately. Ye and his coworkers [15] proposed a novel online deformation method for pathological site tracking. In this method, the stomach's motion was regarded as a local affine transformation. To the authors' knowledge, a local affine assumption is not able to ensure both reliability and consistency in the modeling of the system. In other studies, a force-position sensor or laser transmitter was incorporated in the endoscope to aid the endoscopist in lesion tracking [16, 17]; nevertheless, retargeting previous biopsy sites is still challenging as the sensor's accuracy may be impaired by other medical devices (e.g., pacemakers). In recent years, some atlas-based image registration approaches have been introduced to solve the elastic registration of MRI brain images [18–20]. Those approaches built an atlas based on a large number of template images; the constructed atlas could then be used as a platform for the registration process. However, no related techniques have been proposed to actualize endoscopic image registration due to the image variability inherent to endoscopy.

In this paper, a computer aided endoscopic target tracking method is proposed. This noninvasive method does not require extra instruments, conferring significant advantages in comparison to other approaches. Moreover, this system can predict image registration and track the target lesions for an arbitrary gastroscopic image sequence, even if the deformation between two frames is large. This was accomplished by estimating the similarity of a large number of preobserved sequences of gastroscopic images. With the assumption that the stomach's deformation is not large between very similar frames, the registration between similar frames is treated as a rigid transformation. A gastroscopic image graph is then constructed. In the graph, every frame is designated as a node; the rigid-transformed frames are directly connected nodes and rigid matrixes represent the edges between direct connected nodes. For indirectly connected nodes, the registration estimation is treated as a graph search problem, indicating that nonrigid deformation registration can be estimated by many local rigid transformations. In this paper, a mature graph search method known as Dijkstra's Algorithm was adopted to ascertain the optimal pathway in the graph and determine the deformation model. To describe it clearly in this paper, the frames where the targets were originally marked by the endoscopist are designated as reference images, while the following frames where the targets are tracked are labeled as moving images. During the procedure, in order to determine the targets' locations, the reference images and moving images are matched with the nodes in the graph, and afterwards, the desired deformation can be described as the pathway between corresponding graph nodes. Subsequently, the marked targets are tracked based on the estimated deformation model through the use of the pathway search method.

It should be noted that the construction of the graph is required only once; thus, the computational cost for a tracking task depends on matching the reference and moving images with graph's nodes and searching the proper pathway to calculate the deformation. There are two processes used to improve the performance: (1) the proposed graph is organized as a hierarchical structure and makes the matching process both effective and fast; (2) the edge's rigid matrix provides a matching direction which can direct the tracked images to seek out the most suitable node in the graph. Experiment results demonstrate that the registration can be performed in real time.

## 2. Methods

The Methods section is formatted as follows: basic registration method selection, which is fundamental for estimating node similarity and constructing gastroscopic graphs, is introduced in Section 2.1. In Section 2.2, a detailed scheme covering similarity estimation and graph representation is presented.  Section 2.3 elucidates the process of matching reference and moving images with the graph. Finally, in Section 2.4, the graph search algorithm is demonstrated.

### 2.1. Basic Registration Method Selection

In the graph, we assume that the similar images can be connected directly; thus, a reasonable registration method should be adopted to estimate the similarity before graph construction. Although a wide range of registration methods have been developed over recent decades [21, 22], there is an absence of a gold standard for the evaluation of gastroscopic sequence registrations. It is essential to validate the feasibility of current registration methods to determine the most suitable feature detector as the gastroscopic images' similarity estimation standard.

**Figure 1:**
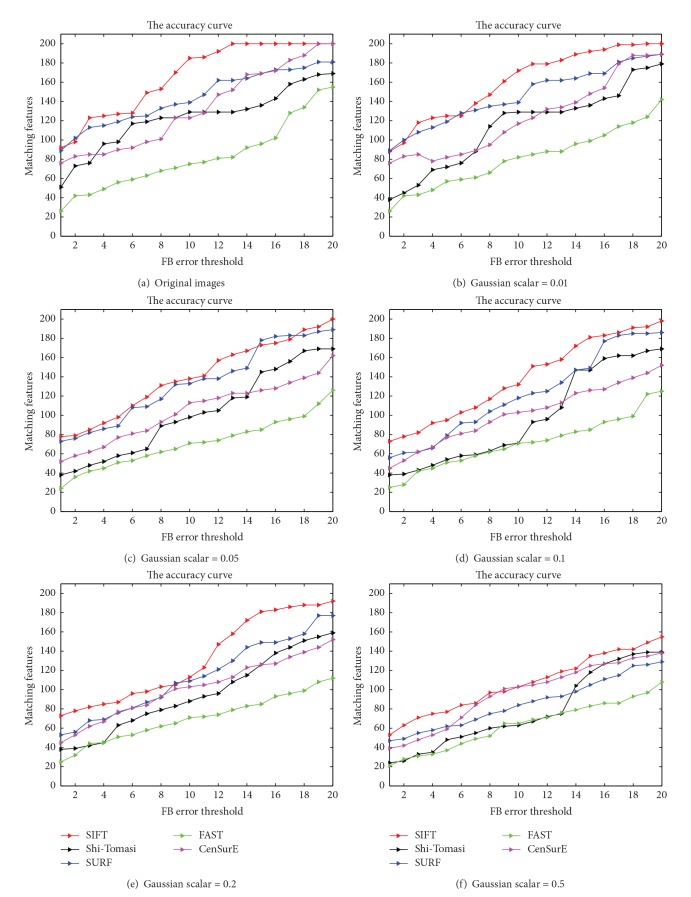
Five tested method registration results. From (a) to (f), the registration methods were applied to original gastroscopic and noise images (the Gaussian noise scalar varies from 0.01 to 0.5). For each registration method, the initial detected feature number was 200; thus, the ideal matching features' number was also 200. In the figures, the color curves represent the number of the detected features whose FB error is smaller than the corresponding FB error threshold (unit: pixels).

In order to employ the most suitable estimation method, five common, widely used feature detectors (Shi-Tomasi, SIFT, FAST, SURF, and CenSurE) [23–27] were selected. An estimation framework, known as forward-backward, was applied [28] to evaluate the selected methods' accuracy and robustness. This framework detects and matches image features from the first frame to the last frame, and afterwards, features are tracked backward to the first frame. If a feature is perfectly detected and matched, it should return to its initial location in the first frame. Otherwise, the distance between the original location and tracked location is considered forward-backward error (FB error). In this paper, the five selected registration methods were used to evaluate gastroscopic images. Moreover, to estimate the robustness of these methods, a series of standard Gaussian noises with different scalars (standard deviation) were added to the original gastroscopic images, and the FB error was regarded as criteria for accuracy and robustness. The evaluation results are shown in Figure 1.

As we can see from Figure 1, for original images (Figure 1(a)), all the features' FB distance detected by SIFT was smaller than 13 pixels, and almost 60% of the features had a FB error smaller than 5 pixels, indicating SIFT's higher accuracy in comparison to the other methods. Furthermore, from Figures 1(b) to 1(f), we can see SIFT also demonstrated a better performance in robustness than the other tested methods for gastroscopic images.

Consequently, in this paper, we adopted SIFT as the basic registration method to estimate the similarity in gastroscopic images. In practice, we found the following two simplified heuristic strategies for SIFT worked well in our methods.

1. The SIFT descriptor was detected in a multiscale space. Generally, the main computational costs depend on establishing the multiscale space. Some researchers have found that the performance of SIFT improves exponentially with the reduction of the image scale [29]. In a real gastroscopy procedure, the endoscope's focal length is fixed, and the motion range of the endoscope is limited by the narrow gastric cavity; thus, the image scale cannot change drastically. In our research, almost 90% of features in the gastroscopic sequences were concentrated between 1 and 3 image scale degrees. As a result, the registration method in this paper detects sift features between image scales of 1 and 3.
2. The original SIFT was designed as a vector of 128 dimensions to make the descriptor robust to changes in brightness, scale, and rotation. To accelerate the performance, this paper simplifies the descriptor to 32 dimensions. Furthermore, to the analysis resistant to the influences of variances in illumination, the elements of the vectors bigger than 0.4 were normalized to 1. It should be noted that the normalized SIFT vectors did not eliminate the illumination but suppressed the effects, which could be recognized in the similarity estimation method discussed in the following section.

### 2.2. Graph Construction

The construction of the gastroscopic graph should adhere to two conditions.

1. Every image node should be connected with every other node directly or indirectly because isolated nodes are useless in estimation of deformation and target tracking.
2. The number of connected edges should be as small as possible, which is beneficial for searching the most reasonable pathway in the graph as quickly as possible.

The similarity estimation should be defined to determine whether the gastroscopic nodes were connected directly or indirectly. In this paper, the similarity of the two nodes, image *i* and image *j*, was defined as

(1) $$\begin{array}{c} S_{ij}=\frac{M_{p}}{\max\left({Fp}_{i},{Fp}_{j}\right)}. \end{array}$$

In (1), *Fp*_*i*_ and *Fp*_*i*_ denote the feature points detected by the simplified SIFT. *M*_*p*_ denotes the matched points. *S*_*ij*_ varies from 0 to 1, with *S*_*ij*_ = 1 and *S*_*ij*_ = 0 being indications of highly consistent or differing images, respectively. Here, we assume the image nodes that qualify the following condition can be connected:

(2) $$\begin{array}{c} S_{ij}>h. \end{array}$$

In this paper, we utilized an iterative process to determine the optimal *h*. It can be described as the following steps.

*Step 1*. Hundreds of image samples from real gastroscopic image sequences were selected randomly and each of the images was clustered with other images. Subsequently, a series of similarity was calculated, assuming *B*_low_ as *Min*⁡(*S*_*ij*_) and *B*_high_ as *Max*⁡(*S*_*ij*_). 

*Step 2*. Calculate the initial *h* as

(3) $$\begin{array}{c} h=B_{\text{high}}-\lambda \left(B_{\text{high}}-B_{\text{low}}\right). \end{array}$$

Here, *λ* = 0.1.

*Step 3*. The affine matrix for the matched image pairs was estimated under the condition: *S*_*ij*_ > *h*. The affine matrix was calculated by the RANSAC algorithm [30] and denoted by *H*_*ij*_ and *H*_*ji*_. Image *j* was warped to image *i* by an affine transform, and the normalized cross correlation (NCC) was utilized for validating the similarity assumption:

(4) $$\begin{array}{c} {\text{Dif}}_{H}=\frac{\sum_{x=0}^{\text{width}}\sum_{y=0}^{\text{height}}I\left(x,y\right)J\left(H\left(x,y\right)\right)}{\sum_{x=0}^{\text{width}}\sum_{y=0}^{\text{height}}I\left(x,y\right)\sum_{x=0}^{\text{width}}\sum_{y=0}^{\text{height}}J\left(H\left(x,y\right)\right)}. \end{array}$$

The more closely the Dif_*H*_ approximates to 1, the higher the similarity of the images is, and the more reasonable the affine assumption is. The experimental results show that if Dif_*H*_ > 0.8, the affine transformation is reasonable.

*Step 4*. Whether all image pairs satisfy *S*_*ij*_ > *h* under the condition of Dif_*H*_ > 0.8 was evaluated. If not, *B*_low_ was set as *h*, and Steps 2 and 3 were repeated. Otherwise, if conditions were satisfied, the algorithm exited and returned the optimal *h*.

NCC was eschewed in favor of the simplified SIFT detector as the basic registration solution due to its lower computational burden.

After calculating the optimal *h*, we assumed the graph as a matrix:

(5) $$\begin{array}{c} G={\left[g_{ij}\right]}_{N*N}. \end{array}$$

In (5), *g*_*ij*_ denotes the pathway weight from node *i* to node *j*. *N* denotes the number of the elements in the graph, and the weight can be represented as

(6) gij={(Sij,Hij),Sij>h,(0,0),otherwise,gji={(Sji,Hji),Sji>h,(0,0),otherwise.

Here, *H*_*ij*_ represents the affine transform matrix from node *i* to node *j* and is calculated by RANSAC algorithm. Obviously, *H*_*ij*_ = *H*_*ji*_^−1^ and *S*_*ij*_ = *S*_*ji*_.

### 2.3. Registration with Graph

**Figure 2:**
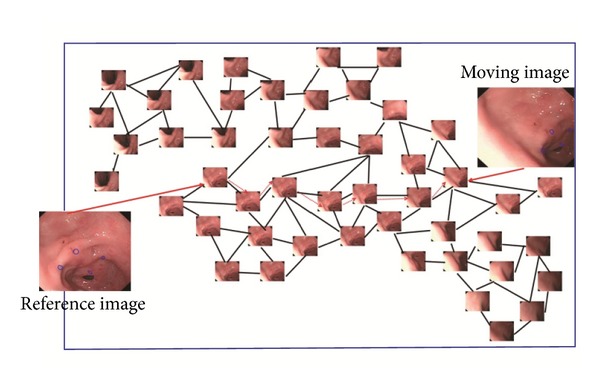
Workflow of registration with the graph. The black lines indicate the nodes that can be connected directly. The red solid lines indicate the matching process of reference image and moving image to seek out the most similar nodes in the graph. The red dash lines indicate the pathway search process. The target tracking result is displayed as blue region on the reference image and moving image.

During a gastroscopy, the endoscopist marks a region of concern on the reference image, and the region is retargeted on the following moving images. In this case, the reference image and moving image should be matched with the graph to determine their corresponding nodes. Subsequently, the pathway is identified from the graph, as well as the deformation model between the reference image and moving image. Finally, the desired region is transformed on the moving image.  Figure 2 describes the registration workflow with the graph.

The pathway search issue is explicated in Section 2.4. We emphatically describe the process of seeking out the most similar nodes in this section.

Here, we utilized NCC to ascertain the most suitable node in the system. Because the captured gastroscopic images vary largely over the course of the procedure, the graph construction required numerous preobserved images. For example, in our experiment, almost 40,000 images were compiled to construct the graph (endoscope model Olympus GIF-QX 260). Identification of the most suitable node requires analysis of each candidate individually at considerable computation cost. Two processes were enacted to ensure the fidelity of performance of the analysis.

1. We divided the graph into subgraphs according to the anatomic sites (e.g., angularis, antral lesser curvature, antral greater curvature, antral posterior wall, and antral anterior wall). During the procedure, the reference image (or moving image) matches with a certain node in the graph randomly; if Dif_*H*_ < 0.2, we consider that the reference image (or moving image) cannot be grouped to a suitable node in the current subgraph, and a node from another subgraph is selected in the subsequent matching process.
2. In the graph, the connected nodes represent their relationship as an affine matrix, which not only provides a solution for estimating the refinement deformation, but also offers direction for targeting the most similar node in the graph (e.g., the translation and rotation from node *i* to node *j*). When matching reference image (or moving image) with a certain node *n*_0_, an estimated affine transform is calculated as *H*_in_0__. Supposing the nodes that connect with *n*_0_ are recorded as [*n*_1_, *n*_2_, *n*_3_,…], the affine relationship is represented as an affine matrix vector: [*H*_*n*_1_*n*_0__, *H*_*n*_2_*n*_0__, *H*_*n*_3_*n*_0__,…]. Before subsequent matching, *H*_in_0__ is compared with all the elements in the affine matrix vector, and the most similar element indicates the corresponding node that has the most consistent movement with the reference image (or moving image). Therefore, this corresponding node is considered the matching node in the successive matching process.

### 2.4. Pathway Determination

After designating reference images and moving images to nodes within the graph, the desired deformation registration should be regarded as a pathway search problem. Because (6), the edge's weight is represented as the similarity of the affine transformation between directly connected nodes, the searched pathway should have the most similarity under the corresponding affine transform.

In the field of computer science, Dijkstra's algorithm [31] is a classic pathway search method; this method generates the shortest pathway tree to solve the single-source, shortest path search-problem using nonnegative edge path costs. Many medical issues have been addressed utilizing this algorithm. For instance, Ehrhardt et al. took advantage of Dijkstra's algorithm to estimate lung motion [32]; additionally, Pantazis et al. and Liu et al. used it to register MRI cortical structures [33, 34]. In this paper, this algorithm was applied to estimate the most reasonable pathway in the gastroscopic graph. Assume the reference image is *i*, and its corresponding node is *n*_*i*_, while the moving image is *j*, and its corresponding node is *n*_*j*_. The pathway search algorithm can be described as follows.

*Step 1*. Image *i* and image *j* are matched directly by (1), and the affine transform matrix is obtained as *H*_*ij*_′ and *H*_*ji*_′.

*Step 2*. Assuming node *k*(*n*_*k*_) is connected with node *i*, the edge weight between them can be calculated as follows:

(7) $$\begin{array}{c} {EW}_{n_{i}n_{k}}=\text{SSD}\left(H_{n_{k}n_{i}}\left(\text{node}\;k\right),H_{n_{j}n_{i}}\left(\text{node}\;j\right)\right). \end{array}$$

In (7), node *k* and node *j* are warped to image *i*, and the sum of the squared difference (SSD) is applied to estimate the edge weight. The smaller the *EW*_*n*_*i*_*n*_*k*__, the higher the probability that node *k* is regarded as a candidate in the desired pathway.

*Step 3*. Applying Dijkstra's algorithm to determine the pathway, the most reasonable pathway has the minimum sum of edge weight.

*Step  4*. Assuming the calculated pathway is [*n*_1_, *n*_2_, *n*_3_,…, *n*_*j*−1_, *n*_*j*_], the estimated deformation between image *i* and image *j* can be represented as

(8) $$\begin{array}{c} H_{ij}=H_{n_{i}n_{1}}H_{n_{1}n_{2}}H_{n_{2}n_{3}},\ldots ,H_{n_{j-1}n_{j}}. \end{array}$$

Consequently, the target region in reference image *i* can be retargeted in the moving image *j* by (8).

## 3. Experimental Results

### 3.1. Phantom Experiment

**Figure 3:**
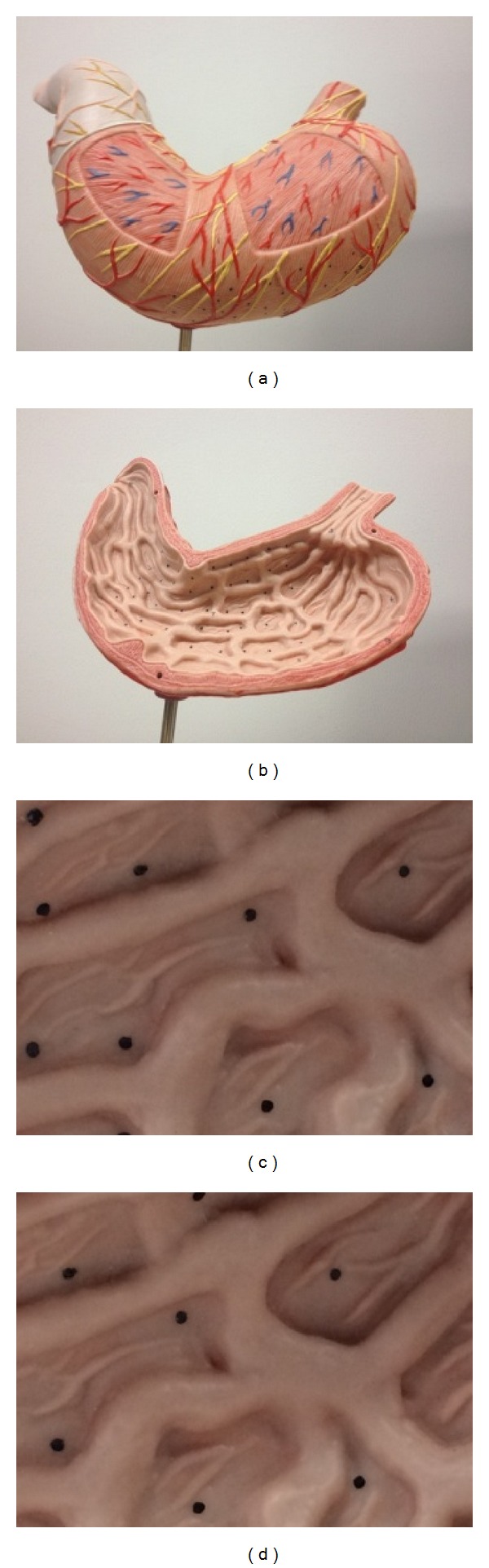
(a)-(b) show the phantom model, and (c)-(d) are images captured by the endoscope.

The proposed graph was tested on both the stomach phantom and* in vivo* data. In the phantom experiment, an Olympus QX 260 endoscope was used to capture a gastroscopy image sequence that included almost 23,000 frames which were then utilized to construct the graph. The frame resolution was 560∗480, and the graph construction process took 3 hours to complete. Several markers were labeled on the phantom model's surface, which were used for simulating the traditional biopsy process (see Figure 3).

**Figure 4:**
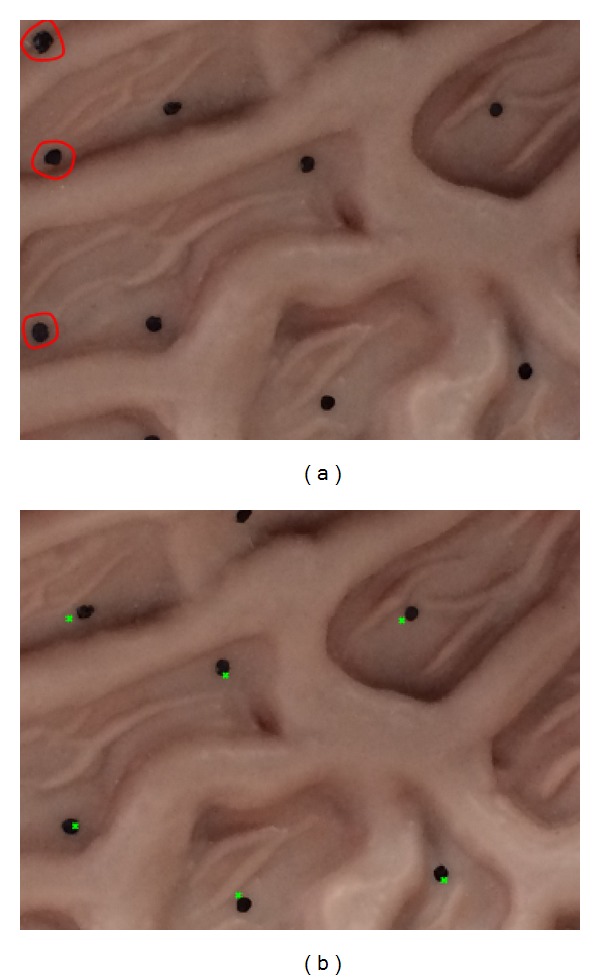
Phantom experiment results. (a) is the reference image, and (b) is the moving image; the time interval between them was 2.8 s in the captured sequence. The results show that six markers were retargeted on the moving images, and three markers in the reference images were missing, marked with red circle in (a), which is considered a reasonable discrepancy. The mean accuracy was 0.78 mm, and the variance of the accuracy was 0.142.

During the experiment, the markers were considered as global truth to determine the accuracy of the graph. The distance between the green markers was the retargeted results, and its corresponding global truth was regarded as the accuracy (see Figure 4).

### 3.2. Clinical Experiment

In the subsequent section, three real gastroscopic images sequences were captured from the angularis, antral structures, and stomach body in real gastroscopy procedures. All the volunteers who participated in the study agreed to the written consent of experimental evaluation and the follow-up medical data analysis. The study protocol conformed to the ethical guidelines of the 1975 Declaration of Helsinki (6th revision, 2008) and was approved by the ethics committee of Sir Run Run Shaw Hospital prior to initiating this study. Additionally, the gastroscopy examination was designed by an experienced clinician and performed by a skilled endoscopist in accordance with the conventional clinical protocol. The Olympus QX 260 endoscope was used; the frame rate of the captured image sequence was 25 f/s, and the resolution was 560∗480.

**Figure 5:**
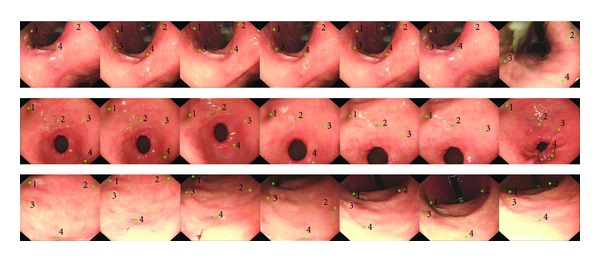
Clincal experiment results. The first row is the retargetting results of the angularis, the middle row is antral structures, and the bottom row is the stomach body. The first image in every row is the reference image, where the green circles are the markers created by endoscopist, and the following images display the lesion retargetting results. The lesions were retargeted every 40 frames; thus, the time interval between adjacent frames is 1 second. To distinguish the markers, all the markers were labled by number. Due to the fold and deformation of the gastric internal surface, the target lesions may have been missed during the procedure. From the figures, it is indicated that this method is sensitive to missing markers and can retarget them when they appeared in the follow-up frames.

During the procedure, the endoscopist steered the scope inspecting the gastric internal surface and labeled suspicious lesions with a virtual green circle instead of traditional tattooing techniques; afterwards, the captured frames and lesions' location were transmitted to a workstation (CPU: Intel(R) Core(TM) i5-3570 3.40 GHz, RAM: 16.00 GB, OS: Windows 7(64 bit)), where the lesions' locations were calculated in the subsequent frames based on the constructed graph. The targeting results were shown on the gastroscopic workstation's screen, as shown in Figure 5.

As the* in vivo* procedure lacked the global truth markers, we utilized the forward-backward method to evaluate the accuracy of the tracked lesions. We regarded each lesion marker's location in the tracked frames as a random variable and took advantage of Kullback-Leibler divergence (*D*_KL_) to measure the similarity of the distributions:

(9) $$\begin{array}{c} D_{\text{KL}1}\left(P_{\text{backward}}\left(x\right)||P_{\text{forward}}\left(x\right)\right)=\underset{x\in X}{\sum }\frac{P_{\text{backward}}\left(x\right)}{P_{\text{forward}}\left(x\right)},\\ D_{\text{KL}2}\left(P_{\text{forward}}\left(x\right)||P_{\text{backward}}\left(x\right)\right)=\underset{x\in X}{\sum }\frac{P_{\text{forward}}\left(x\right)}{P_{\text{backward}}\left(x\right)}. \end{array}$$

In (9), *P*_forward_ and *P*_backward_ denote the lesion marker's distribution in the forward and backward procedure. As the definition of Kullback-Leibler divergence, if the target result is accurate, the distribution of the lesion in the forward procedure is very similar to the distribution in the backward procedure. In this case, the *D*_KL1_ and *D*_KL2_ were small and of the same order. In this paper, we regarded a lesion's trajectory as accurate only when *D*_KL1_ < 0.1 and *D*_KL2_ < 0.1.

In Table 1, the endoscopist marked 100 lesions on the angularis, antrum, and stomach body, respectively. The 300 lesions were then evaluated using forward-backward method. The accuracy percent, mean, and variance indicate the percent of lesions whose divergence value was smaller than 0.1, average FB error of lesion location, and fluctuation, respectively. The diameter of the biopsy forceps was 0.8 cm, which provided a gold standard for converting pixels to millimeters.

The clinical experimental results demonstrated that the accuracy at the angularis was 6.3 ± 2.4 mm, at the antrum was 7.6 ± 3.1 mm, and at the stomach body was 6.9 ± 1.6 mm. The mean accuracy was 7.31 mm. Because forceps diameter was used as a global truth in this process, this new methodology had the similar precision to traditional tattooing protocols. In order to further compare these techniques, we tested the operation time of this method and tattooing.

In Table 2, the operation time was divided into two components. The marking time indicates the time spent on creating the marking during gastroscopy. For tattooing, it included invasive operation and ink injection; for the graph method, the reference image was mapped with the graph. Additionally, the targeting time shows the computational time necessary for targeting the markers in the follow-up frames. Obviously, for tattooing, the targeting time was zero due to the physical symbolic nature of the tattoo; for graph method, including matching moving images with the graph and deformation registration determination.

Because tattooing was a cumbersome operation, even skilled endoscopists needed more than 10 s to mark the lesion with ink. Moreover, the risk of injection failure might occur, and, in this case, the endoscopists have to make the injection more than one time. In contrast, graph based marking method required less time (12 ms) than lesion tattooing. The targeting time for the graph method was almost 50 ms. To realize lesion tracking in real-time during gastroscopy, we calculated the lesion's position every three frames.

## 4. Discussion and Conclusion

In this paper, a target tracking method for gastroscopic image sequence was proposed. The proposed method can be applicable for clinical biopsy procedure. Comparing with traditional biopsy method, this method is noninvasive and does not require additional instruments. This method also provides a solution for postoperative lesion review and intraoperative navigation in the followups, which will be validated with further experimentation in the future.

The experiment results demonstrate that the method's accuracy and operation time were satisfactory for clinical practice. Compared to traditional lesion surveillance methods, the marker created by this method is recorded by the workstation, a platform where stability of stored information is not sensitive to time, unlike ink-based targeting. Additionally, this method's technical operation had a shallow learning curve for endoscopists and had a lower risk of operation failure.

Due to the mechanics and movement of the intestinal system, variety in gastroscopic images, representation varies largely; these obstacles necessitate specific solutions for a graphical representation of image sequences of the intestinal tract. We constructed an independent graph for a corresponding gastroscopic device (e.g., Olympus QX 260). The experimental results show that this methodology is viable for the targeting procedure. Because the endoscope moves flexibly in the stomach, the reference images and moving images may not be categorized to appropriate nodes in the current graph. In the future, a graph update scheme will be considered to extend the graph for further tracking. Additionally, future studies will be focused on validating the graph's accuracy with a large number of patients, endoscopists, and gastroscope devices.

## Figures and Tables

**Table 1:** The accuracy of the retarget process.

	Accuracy percent	Mean (mm)	Variance (mm)
Angularis	0.93	6.3	2.4
Antrum	0.87	7.6	3.1
Stomach body	0.95	6.9	1.6

**Table 2:** Operation time comparison.

	Tattooing (ms) (marking/targeting)	Graph (ms) (marking/targeting)
Angularis	1758/0	12/47
Antrum	1372/0	12/42
Stomach body	1231/0	12/45
